# Supplementary material for: Live imaging and conditional disruption of native PCP activity using endogenously tagged zebrafish sfGFP-Vangl2
Source: Nat Commun. 2022 Sep 23;13:5598. doi: 10.1038/s41467-022-33322-9 (PMC9508082; doi:10.1038/s41467-022-33322-9)
Supplement: Supplementary file 1 — Supplementary Information [file 41467_2022_33322_MOESM1_ESM.pdf]

SUPPLEMENTARY INFORMATION

Live imaging and conditional disruption of native PCP activity using endogenously tagged zebrafish sfGFP-Vangl2

Maria Jussila[1], Curtis W. Boswell[1,2,3], Nigel W. Griffiths[1], Patrick G. Pumputis[1,2] and Brian Ciruna[1,2,*]

1. Program in Developmental & Stem Cell Biology, The Hospital for Sick Children, 686 Bay Street, Toronto, Ontario, M5G 0A4, Canada.

2. Department of Molecular Genetics, The University of Toronto, Toronto, Ontario, M5S 1A8, Canada.

3. Current address: Department of Genetics, Yale University School of Medicine, New Haven, CT 06510, USA.

* Corresponding author. Email: ciruna@sickkids.ca

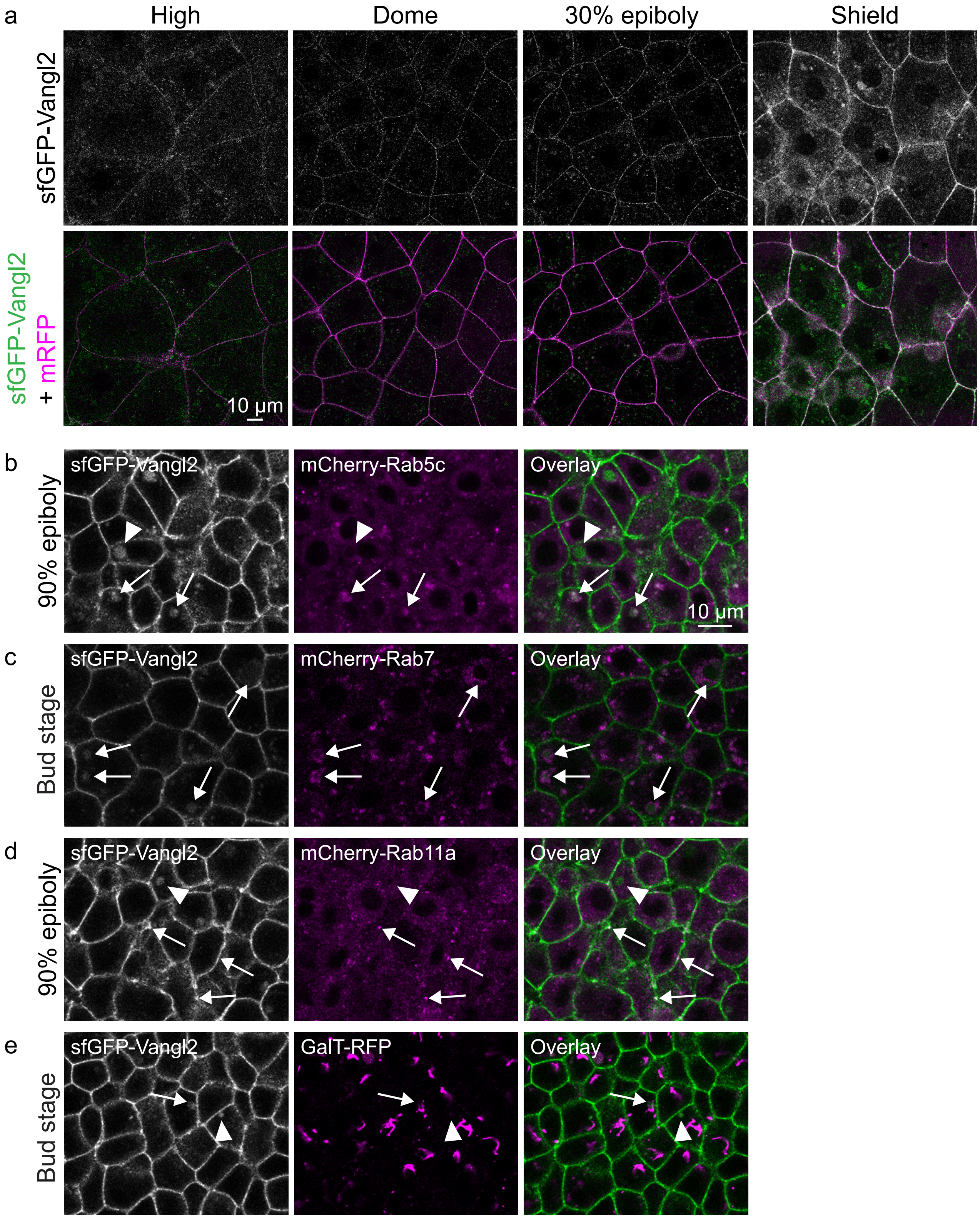

Supplementary Figure 1. Membrane-localization and endosomal recycling of sfGFP-Vangl2 during early zebrafish embryogenesis

(a) Live confocal images of EVL cells of *vangl2^{sfGFP}* embryos at blastula and early gastrula stages (n = 6 for each stage). Embryos were injected with mRNA coding for a membrane-localized monomeric RFP (mRFP) reporter. All sfGFP images were acquired using identical settings.

(b-e) Live confocal images of ectodermal cells between dorsal and lateral domains in 90% epiboly or bud staged *vangl2^{sfGFP}* embryos at injected with mRNA coding for mCherry-Rab5c (b), mCherry-Rab7 (c), mCherry-Rab11a (d) or GalT-RFP (e) reporters (n = 4 for each marker). Arrows point at cytoplasmicVangl2 puncta in close proximity to respective reporters, and arrowheads point at isolated Vangl2 puncta.

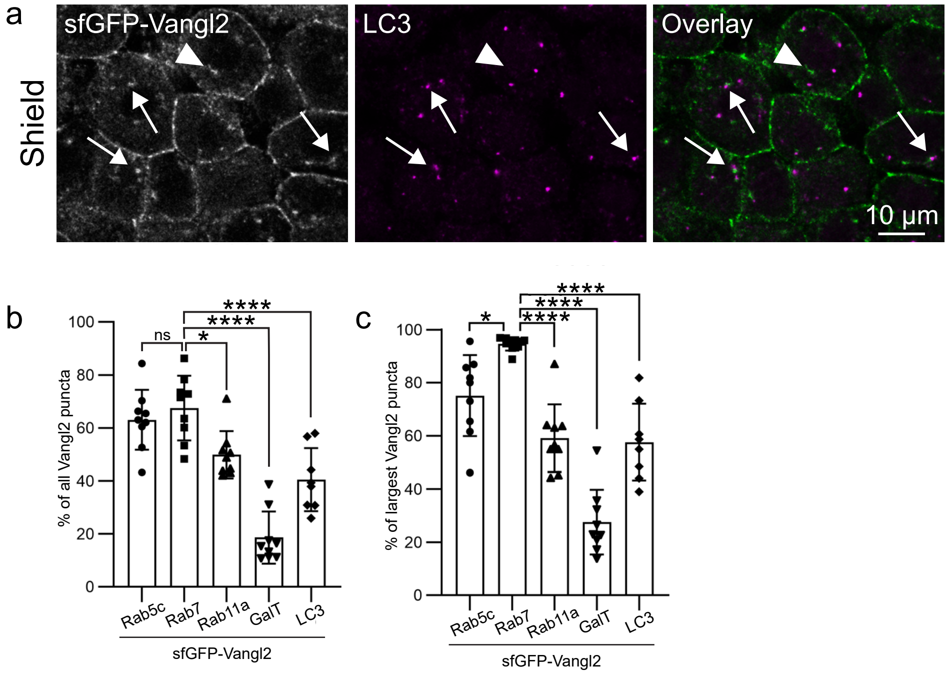

Supplementary Figure 2. sfGFP-Vangl2 is not primarily targeted to autophagosomes
(a) Confocal image of a representative shield staged *vangl2*^{sfGFP} embryo showing immunostaining against LC3 protein (n = 4). Arrows point at cytoplasmic Vangl2 puncta in close proximity LC3, and arrowhead at isolated Vangl2 puncta.
(b) Quantification of the percentage of cytoplasmic sfGFP-Vangl2 puncta touching puncta positive for markers imaged in Fig. 1 f-i. Statistical analysis was performed using one-way ANOVA with Tukey's multiple comparisons test (**P*=0.0112, *****P*<0.0001). Rab5c to GalT N = 3 embryos, 3 areas analyzed per embryo, LC3 N = 4 embryos, 2 areas analyzed per embryo. Data are presented as mean ±SD. Source data are provided as a Source Data file.
(c) Quantification of the percentage of cytoplasmic sfGFP-Vangl2 puncta larger than 1.5 µm^3 touching puncta positive for markers imaged in Fig. 1 f-i. Statistical analysis was performed using one-way ANOVA with Tukey's multiple comparisons test (**P*=0.0141, *****P*<0.0001). Rab5c to GalT N = 3 embryos, 3 areas analyzed per embryo, LC3 N = 4 embryos, 2 areas analyzed per embryo. Data are presented as mean ±SD. Source data are provided as a Source Data file.

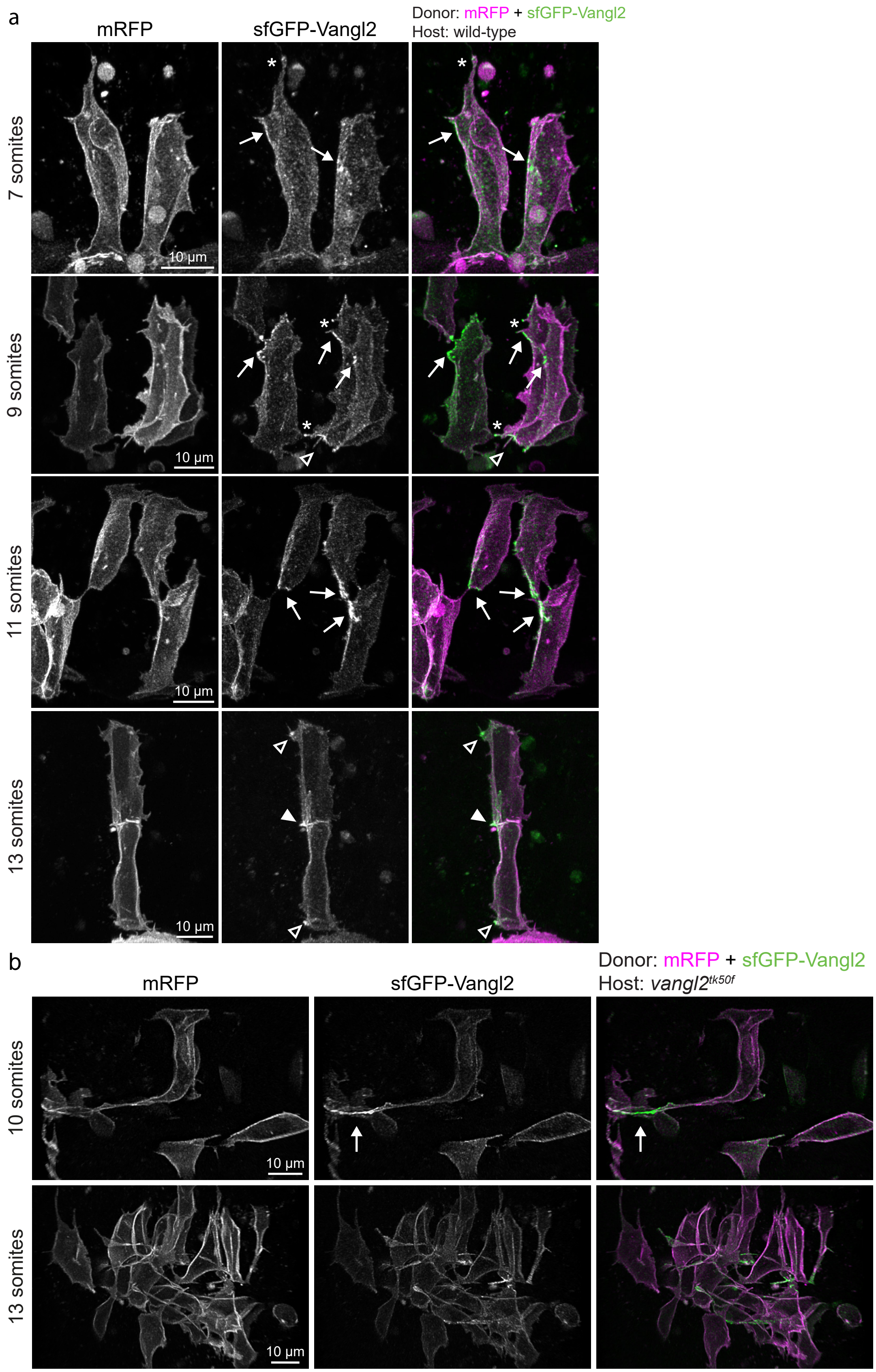

a
mRFP
sfGFP-Vangl2
Donor: mRFP + sfGFP-Vangl2
Host: wild-type
7 somites
9 somites
11 somites
13 somites
10 µm
b
mRFP
sfGFP-Vangl2
Donor: mRFP + sfGFP-Vangl2
Host: vangl2tk50f
10 somites
13 somites
10 µm

Supplementary Figure 3. Vangl2 is enriched on anterior membranes of neuroepithelial cells and is membrane-localized but not polarized in a PCP deficient environment

(a) Live confocal images of membrane-RFP and sfGFP-Vangl2 localization in the developing spinal cord of chimeric embryos (*mRFP* mRNA-injected $vangl2^{sfGFP}$ cells transplanted into wild-type hosts) at progressive stages of neural tube morphogenesis. Representative images of cells analysed in Fig.2 d-e. Maximum intensity projections are shown, anterior is to the left. Arrows point at Vangl2 enrichment on cell membranes, arrowheads at apical membranes at the neural midline, open arrowheads at Vangl2 enrichment on basal membranes, and asterisks at Vangl2-positive cell protrusions.

(b) Live confocal images of mRFP and sfGFP-Vangl2 localization in transplanted $vangl2^{sfGFP}$ neuroepithelial cells within $vangl2^{tk50f}$ mutant hosts, at 10-somite and 13-somite stages of development. Representative images of cells analysed in Fig.3 b-c. Maximum intensity projections are shown, anterior is to the left. Arrow points at Vangl2 enrichment to a membrane protrusion.

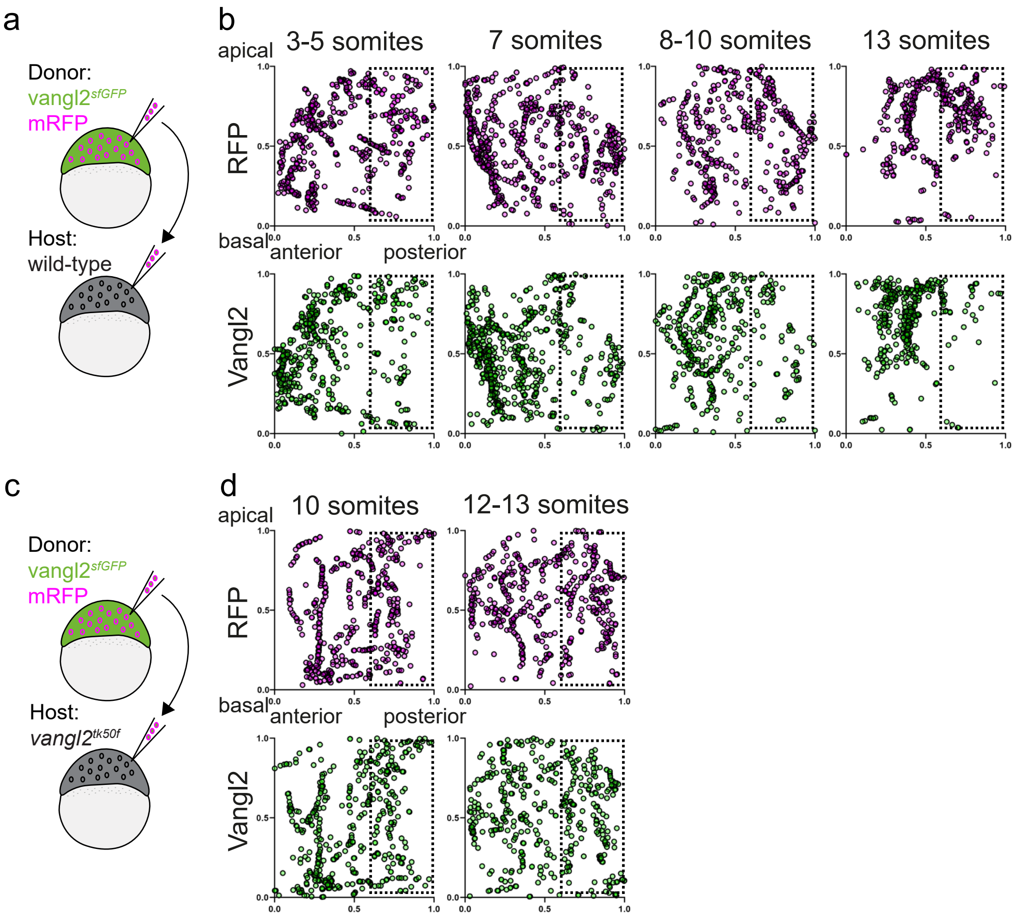

Supplementary Figure 4. Quantification of Vangl2 localization shows that it is anteriorly enriched and requires functional PCP for planar polarization.

(a) A schematic illustrating cell transplantation from a membrane-RFP labelled $vangl2^{sfGFP}$ donor embryo into a wild-type host embryo at sphere stage.

(b) Scatter plots showing the distribution of the brightest sfGFP-Vangl2 and membrane-RFP spots along anterior-posterior (X) and apical-basal (Y) axes of $vangl2^{sfGFP}$ neuroepithelial cells, quantified at four consecutive stages of neural tube morphogenesis. Data from multiple transplanted $vangl2^{sfGFP}$ cells was pooled (3-5 somites, n = 7; 7 somites, n = 7; 8-10 somites, n = 6; 13 somites, n = 5) and axial lengths normalized to 1. Values are the same as plotted along individual axes in Fig. 2. Dashed boxes highlight the differences of membrane-RFP and sfGFP-Vangl2 localization in the posterior part of cells. Details of the quantification can be found in the Methods section. Source data are provided as a Source Data file.

(c) A schematic illustrating cell transplantation from a membrane-RFP labelled $vangl2^{sfGFP}$ donor embryo into a $vangl2^{tk50f}$ host embryo at sphere stage.

(d) Scatter plots showing the distribution of the brightest Vangl2 and membrane-RFP spots along anterior-posterior (X) and apical-basal (Y) axes of $vangl2^{sfGFP}$ neuroepithelial cells in a $vangl2^{tk50f}$ mutant host, quantified at two consecutive stages of neural tube morphogenesis. Data from multiple transplanted $vangl2^{sfGFP}$ cells was pooled (10 somites, n = 7; 12-13 somites, n = 9) and axial lengths normalized to 1. Values are the same as plotted along individual axes in Fig. 3. Dashed boxes highlight the similarity of membrane-RFP and sfGFP-Vangl2 localization in the posterior part of cells. Source data are provided as a Source Data file.

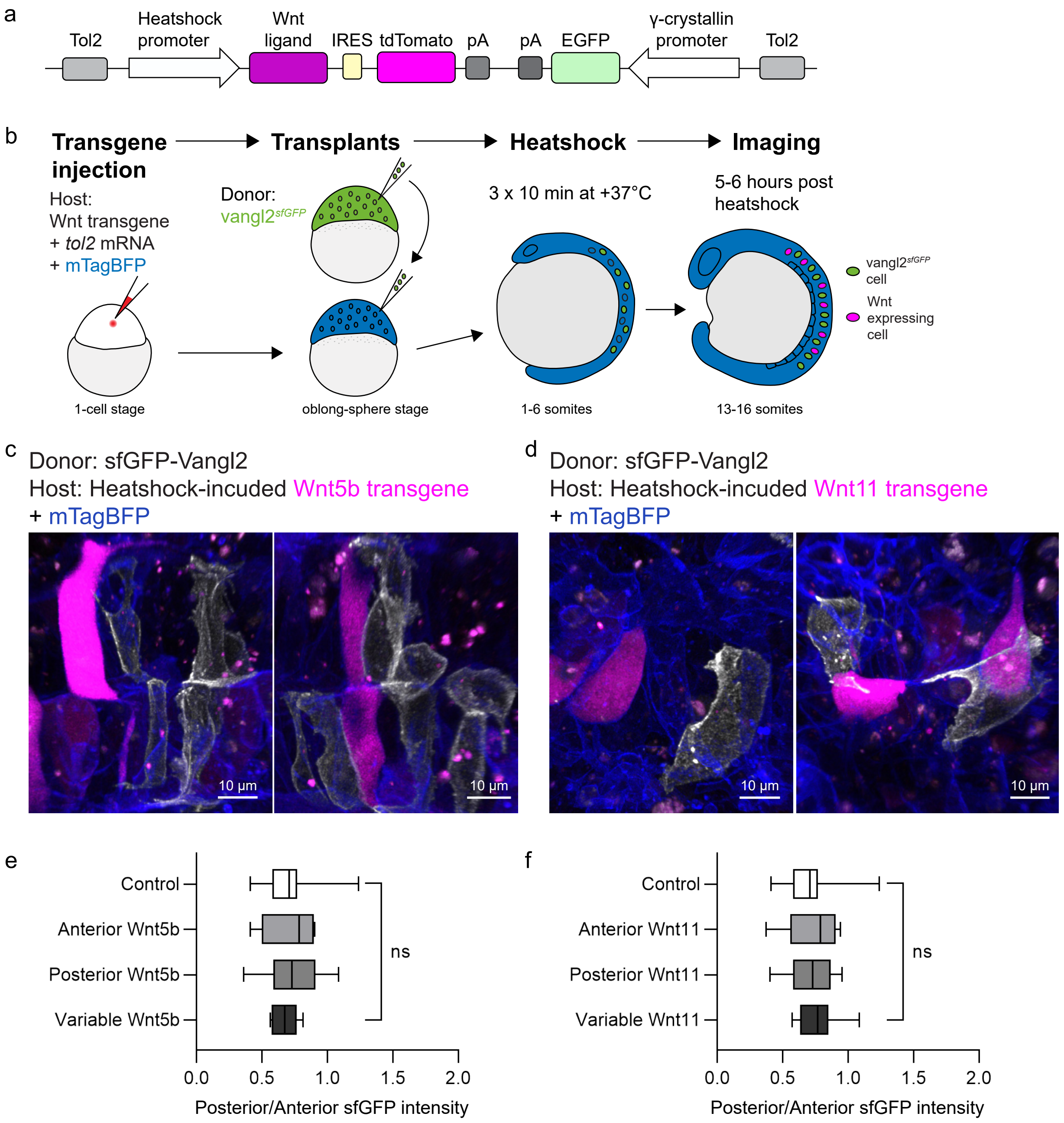

Supplementary Figure 5. Exogenous sources of non-canonical Wnt ligands fail to instruct or disrupt planar polarity of neuroepithelial cells.

(a) A schematic of heatshock-inducible Wnt expression transgenes.

(b) A schematic of the experimental workflow for generating *vangl2*^{sfGFP} chimeric embryos with mosaic integration of heatshock-inducible *Tg(hsp70::wnt5b-IRES-tdTomato)* or *Tg(hsp70::wnt11-IRES-tdTomato)* transgenes.

(c-d) Live confocal images of *vangl2*^{sfGFP} cells and *wnt5b-* (c) or *wnt11-* (d) expressing cells visualized by tdTomato expression in the developing spinal cord of chimeric embryos (*vangl2*^{sfGFP} cell transplanted into wild-type hosts, previously injected with *mTagBFP-CAAX* and *Tol2* mRNAs plus respective Wnt transgene plasmid DNA) subjected to a heatshock treatment. Maximum intensity projections are shown, anterior is to the left. Representative samples shown for cells quantified in (e-f).

(e-f) Quantification of posterior:anterior sfGFP signal intensity ratio for *vangl2*^{sfGFP} cells in close proximity to *wnt5b-* € or *wnt11-* (f) expressing cells. In e, control n = 22 cells, anterior Wnt5b n = 7 cells, posterior Wnt5b n = 12 cells, variable Wnt5b n = 5 cells. In f, control n = 22 cells, anterior Wnt11 n = 6 cells, posterior Wnt11 n = 7 cells, variable Wnt11 n = 10 cells. Statistical analysis was performed using two-tailed t-test. Whiskers indicate minimum and maximum values, box indicates first and third quartile, and the vertical line median value. Source data are provided as a Source Data file.

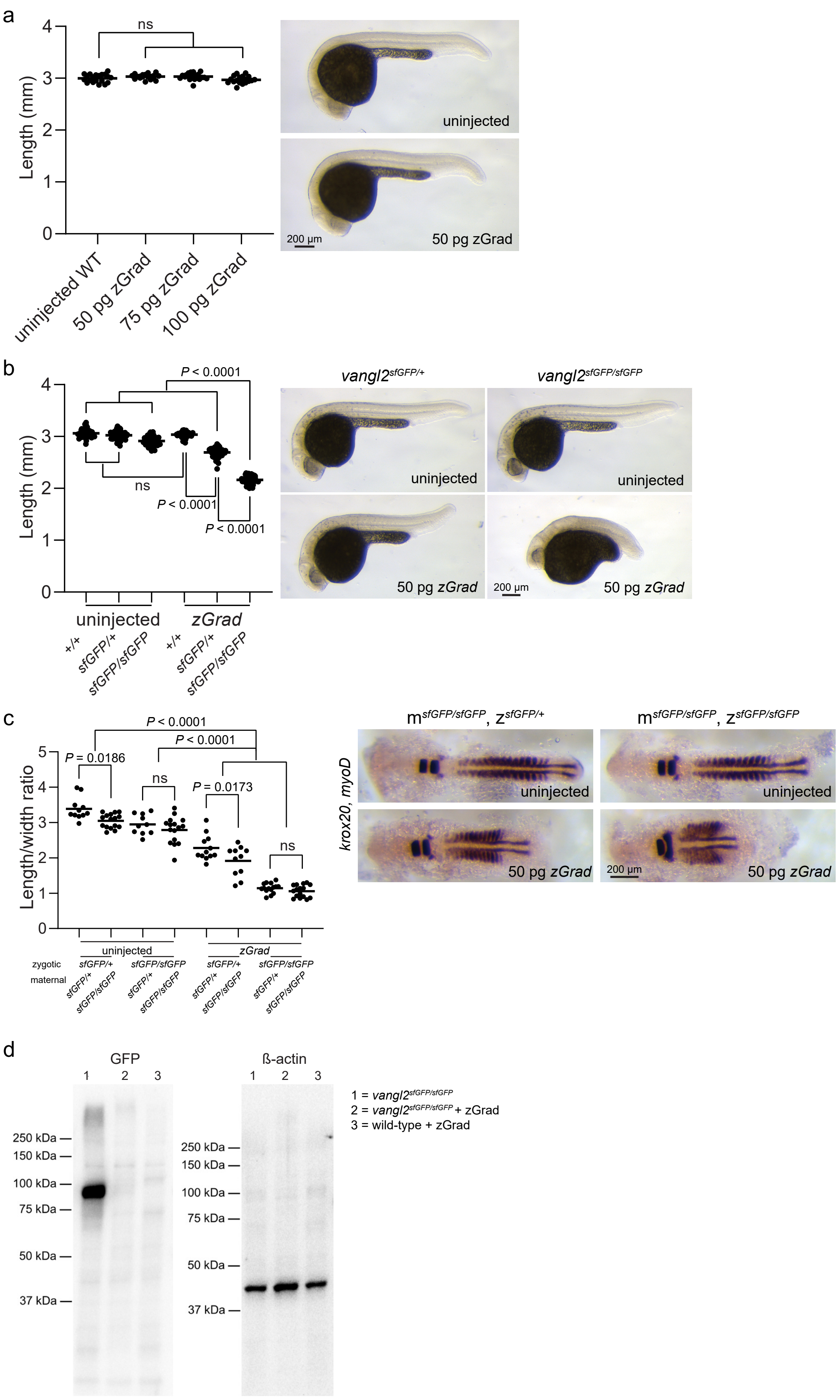

a
ns
Length (mm)
4
3
2
1
0
uninjected WT
50 pg zGrad
75 pg zGrad
100 pg zGrad
uninjected
200 µm
50 pg zGrad
b
P < 0.0001
ns
P < 0.0001
P < 0.0001
Length (mm)
uninjected
zGrad
+/+
sfGFP/+
sfGFP/sfGFP
vangl2sfGFP/+
vangl2sfGFP/sfGFP
uninjected
50 pg zGrad
200 µm
c
P < 0.0001
P < 0.0001
P = 0.0186
ns
P = 0.0173
ns
Length/width ratio
5
4
3
2
1
0
uninjected
zGrad
zygotic
maternal
sfGFP/+
sfGFP/sfGFP
msfGFP/sfGFP, zsfGFP/+
msfGFP/sfGFP, zsfGFP/sfGFP
krox20, myoD
uninjected
50 pg zGrad
200 µm
d
GFP
ß-actin
1
2
3
250 kDa
150 kDa
100 kDa
75 kDa
50 kDa
37 kDa
1 = vangl2sfGFP/sfGFP
2 = vangl2sfGFP/sfGFP + zGrad
3 = wild-type + zGrad

Supplementary Figure 6. Optimizing zGrad GFP-nanobody protein degradation methodologies to disrupt sfGFP-Vangl2 function

(a) Quantification of 24 hpf embryo lengths following *zGrad* mRNA injection into wild-type embryos. Data shows one of two performed experiments. Statistical analysis was performed using one-way ANOVA with Tukey's multiple comparisons test. N = 20 for each condition. Representative uninjected control embryos and 50 pg *zGrad* mRNA-injected embryos are shown in a lateral view. Horizontal line labels mean, and dots represent individual embryo measurements. Source data are provided as a Source Data file.

(b) Quantification of 24 hpf embryo lengths following 50pg *zGrad* mRNA injection into embryos obtained from a $vangl2^{sfGFP/+}$ incross, genotypes are as indicated. Data is pooled together from two experiments. Statistical analysis was performed using one-way ANOVA with Tukey's multiple comparisons test. Control wild-type, n = 60; $vangl2^{sfGFP/+}$, n = 60; $vangl2^{sfGFP/sfGFP}$, n = 56; *zGrad*-injected wild-type, n = 43; $vangl2^{sfGFP/+}$, n = 60; $vangl2^{sfGFP/sfGFP}$, n = 48. Representative uninjected and 50 pg *zGrad* mRNA injected $vangl2^{sfGFP/+}$ and $vangl2^{sfGFP/sfGFP}$ embryos are shown in a lateral view. Horizontal line labels mean, and dots represent individual embryo measurements. Source data are provided as a Source Data file.

(c) Quantification of length/width ratio of *zGrad* mRNA-injected and uninjected control embryos at 15 somite stages. Embryos obtained from crosses between $vangl2^{sfGFP/+}$ and $vangl2^{sfGFP/sfGFP}$ parents; maternal and zygotic $vangl2^{sGFP}$ genotypes are as indicated. Statistical analysis was performed using two-way ANOVA with Tukey's multiple comparisons test. Control n from left to right: n = 11, n = 16, n = 10, n = 16. zGrad n from left to right: n = 12, n = 11, n = 15, n = 16. Representative uninjected and 50 pg *zGrad* mRNA injected $vangl2^{sfGFP/+}$ and $vangl2^{sfGFP/sfGFP}$ embryos from $vangl2^{sfGFP/sfGFP}$ mothers are shown in a dorsal view. *krox20* and *myoD* expression were used to define hindbrain and somitic mesoderm boundaries, respectively, as detected by whole-mount RNA *in situ* hybridization. Horizontal line labels mean, and dots represent individual embryo measurements. Source data are provided as a Source Data file.

d) Uncropped images of the Western blots in Fig. 5b.
