# Supplementary material for: Live imaging and conditional disruption of native PCP activity using endogenously tagged zebrafish sfGFP-Vangl2
Source: Nat Commun. 2022 Sep 23;13:5598. doi: 10.1038/s41467-022-33322-9 (PMC9508082; doi:10.1038/s41467-022-33322-9)
Supplement: Supplementary file 3 — Description of Additional Supplementary Files [file 41467_2022_33322_MOESM3_ESM.pdf]

Description of Additional Supplementary Files

File Name: Supplementary Movie 1
Description: Vangl2 enrichment on anterior membranes is dynamic over time

File Name: Supplementary Movie 2
Description: Vangl2 enrichment on the anterior membrane does not colocalize with filamentous actin

File Name: Supplementary Movie 3
Description: Vangl2 localizes to anterior apical membranes and to basal membrane extensions in dividing cells

File Name: Supplementary Movie 4
Description: Vangl2 anterior polarization is lost during cell division
