# Supplementary material for: Live imaging and conditional disruption of native PCP activity using endogenously tagged zebrafish sfGFP-Vangl2
Source: Nat Commun. 2022 Sep 23;13:5598. doi: 10.1038/s41467-022-33322-9 (PMC9508082; doi:10.1038/s41467-022-33322-9)
Supplement: Supplementary file 8 — Reporting Summary [file 41467_2022_33322_MOESM8_ESM.pdf]

Corresponding author(s): Brian Ciruna
Last updated by author(s): Sep 4, 2022

Reporting Summary

Nature Portfolio wishes to improve the reproducibility of the work that we publish. This form provides structure for consistency and transparency in reporting. For further information on Nature Portfolio policies, see our Editorial Policies and the Editorial Policy Checklist.

Statistics

For all statistical analyses, confirm that the following items are present in the figure legend, table legend, main text, or Methods section.

n/a	Confirmed	
☐	☒	The exact sample size (*n*) for each experimental group/condition, given as a discrete number and unit of measurement
☐	☒	A statement on whether measurements were taken from distinct samples or whether the same sample was measured repeatedly
☐	☒	The statistical test(s) used AND whether they are one- or two-sided<br>*Only common tests should be described solely by name; describe more complex techniques in the Methods section.*
☒	☐	A description of all covariates tested
☐	☒	A description of any assumptions or corrections, such as tests of normality and adjustment for multiple comparisons
☐	☒	A full description of the statistical parameters including central tendency (e.g. means) or other basic estimates (e.g. regression coefficient) AND variation (e.g. standard deviation) or associated estimates of uncertainty (e.g. confidence intervals)
☐	☒	For null hypothesis testing, the test statistic (e.g. *F*, *t*, *r*) with confidence intervals, effect sizes, degrees of freedom and *P* value noted<br>*Give P values as exact values whenever suitable.*
☒	☐	For Bayesian analysis, information on the choice of priors and Markov chain Monte Carlo settings
☒	☐	For hierarchical and complex designs, identification of the appropriate level for tests and full reporting of outcomes
☒	☐	Estimates of effect sizes (e.g. Cohen's *d*, Pearson's *r*), indicating how they were calculated

Our web collection on statistics for biologists contains articles on many of the points above.

Software and code

Policy information about availability of computer code

Data collection	The following sofware was used for image aquisition: Leica LAS X, Leica LAS X Lightning, ChemiDoc MP (Bio-Rad), CTVox SEM software (Bruker)
Data analysis	FIJI version 1.53f51 (ImageJ) and Imaris version 9.9.1 (Bitplane) were used for image analysis and preparing manuscript figures, Imaris Viewer version 9.9.1 (Bitplane), Photoshop release 23.4.2 (Adobe) and Illustrator release 26.4.1 (Adobe) were used to prepare figures, Prism 9 version 9.3.1 (GraphPad) was used for statistical testing.

For manuscripts utilizing custom algorithms or software that are central to the research but not yet described in published literature, software must be made available to editors and reviewers. We strongly encourage code deposition in a community repository (e.g. GitHub). See the Nature Portfolio guidelines for submitting code & software for further information.

Data

Policy information about availability of data

All manuscripts must include a data availability statement. This statement should provide the following information, where applicable:
- Accession codes, unique identifiers, or web links for publicly available datasets
- A description of any restrictions on data availability
- For clinical datasets or third party data, please ensure that the statement adheres to our policy

All quantified data is available as a supplemental Source Data file. Raw imaging files are available upon request due to large size.

Field-specific reporting

Please select the one below that is the best fit for your research. If you are not sure, read the appropriate sections before making your selection.

☒ Life sciences ☐ Behavioural & social sciences ☐ Ecological, evolutionary & environmental sciences

For a reference copy of the document with all sections, see nature.com/documents/nr-reporting-summary-flat.pdf

Life sciences study design

All studies must disclose on these points even when the disclosure is negative.

Sample size	No sample size calculations were performed. Sample size was limited by the number of embryonic and cell samples available and based partially on our previous experience.
Data exclusions	No data were excluded from analysis.
Replication	Biological replicates were conducted and they reproduced all findings. Number of replicates is indicated in the figure legends.
Randomization	Embryos were randomly allocated to control and zGrad mRNA injected groups by randomly dividing and injecting half of a clutch with mRNA at 1 cell stage. In other experiments samples were not randomized as we used genetic mutant and control animals.
Blinding	In embryonic length quantifications, samples that required genotyping were measured prior to genotyping. In other experiments blinding was not possible as obvious phenotypes or fluorescent marker expression were visible.

Reporting for specific materials, systems and methods

We require information from authors about some types of materials, experimental systems and methods used in many studies. Here, indicate whether each material, system or method listed is relevant to your study. If you are not sure if a list item applies to your research, read the appropriate section before selecting a response.

Materials & experimental systems

n/a	Involved in the study	
☐	☒	Antibodies
☒	☐	Eukaryotic cell lines
☒	☐	Palaeontology and archaeology
☐	☒	Animals and other organisms
☒	☐	Human research participants
☒	☐	Clinical data
☒	☐	Dual use research of concern

Methods

n/a	Involved in the study	
☒	☐	ChIP-seq
☒	☐	Flow cytometry
☒	☐	MRI-based neuroimaging

Antibodies

Antibodies used	Anti-LC3A, 1:100, rabbit polyclonal, Novus Biologicals #NB100-2331SS, lot AM; anti-Reissner substance, 1:500, rabbit polyclonal, non-commercial, doi:10.1016/j.cub.2020.04.020; anti-polyglutamylation modification, 1:500, mouse monoclonal, clone GT335, Adipogen Lifesciences #AG-20B-0020-C100, lot A27791601; anti-GFP, 1:2000, rabbit polyclonal, ThermoFisher Scientific #A11122, lot 51527A; anti-beta-actin, 1:500, rabbit polyclonal, Cell Signaling Technology #4967, lot 12; goat anti-rabbit HRP, 1:15000, polyclonal, Abcam #6721, lot GR3357864-9; Goat anti-Rabbit IgG (H+L) Alexa Fluor Plus 594, 1:1000, polyclonal, ThermoFisher Scientific #A32740, lot UG288488; goat anti-Rabbit IgG (H+L) Alexa Fluor Plus 488, 1:1000, polyclonal, ThermoFisher Scientific #A32731, lot UI295697; Goat anti-Mouse IgG (H+L) Alexa Fluor Plus 647, 1:1000, polyclonal, ThermoFisher Scientific #A32728, lot WK331591
Validation	LC3a antibody has been previously used in zebrafish, doi:10.1371/journal.pgen.1003303; anti-Reissner substance has been previously used in zebrafish doi:10.1016/j.cub.2020.04.020; anti-polyglutamylation modification has been validated for immunofluorescent by the manufacturer; anti-GFP has been verified for Western Blot by manufacturer and our GFP degradation experiments further verify specificity; anti-beta-actin has been verified for Western Blot and reactivity in zebrafish by the manufacturer, the goat anti-rabbit HRP and Alexa Fluor secondary antibodies have been validated by the manufacturer

Animals and other organisms

Policy information about studies involving animals; ARRIVE guidelines recommended for reporting animal research

Laboratory animals	Wild-type TU (Tübingen), vangl2 mutant tritk50f and trim209 (Jessen et al. 2002) and transgenic Tg(foxj1a::iCre) (Van Gennip et al. 2018) were used in addition to the lines generated in this study. Reproductive age (2-18 months) female and male zebrafish were used to obtain embryonic samples used in the study.

March 2021

Wild animals	No wild animals were used.
Field-collected samples	No field-collected samples were used.
Ethics oversight	Zebrafish husbandry and experimental protocols were approved by the Hospital for Sick Children's Animal Care Committee, and all protocols were performed in accordance with Canadian Council on Animal Care guidelines.

Note that full information on the approval of the study protocol must also be provided in the manuscript.
